# Longitudinal associations between capacity to be alone, life satisfaction, self‐compassion, anxiety, and depression among Chinese college students

**DOI:** 10.1002/pchj.783

**Published:** 2024-06-23

**Authors:** Qihui Tang, Xinyuan Zou, Shujian Wang, Liang Zhang, Xiangping Liu, Congying Shi, Yanqiang Tao, Yuting Li

**Affiliations:** ^1^ Faculty of Psychology Beijing Normal University Beijing China; ^2^ Beijing Key Laboratory of Applied Experimental Psychology National Demonstration Center for Experimental Psychology Education Beijing China; ^3^ College Students' Mental Health Education Center Northeast Agricultural University Harbin China; ^4^ School of Psychology Nanjing Normal University Nanjing China; ^5^ Department of Chinese Medicine Nursing, School of Nursing Anhui University of Chinese Medicine Hefei China

**Keywords:** anxiety, capacity to be alone, depression, life satisfaction, self‐compassion

## Abstract

Although the world has entered the post‐pandemic period, the mental health and life satisfaction of college students still need to be addressed. However, previous literature has primarily focused on negative variables and has paid little attention to positive variables, such as self‐compassion and the capacity to be alone. Therefore, this longitudinal study aims to investigate the relationships between the capacity to be alone, self‐compassion, life satisfaction, depression, and anxiety among college students. This study analyzed data from 1460 Chinese college students who completed an online survey at two time‐points one year apart. We employed cross‐lagged analysis and constructed longitudinal mediation models to explore the relationships between five variables (i.e., capacity to be alone, self‐compassion, life satisfaction, depression, and anxiety). Our findings indicate that depression and life satisfaction could negatively predict each other over time. Self‐compassion in wave 1 could negatively predict depression and anxiety in wave 2. Higher life satisfaction in wave 1 was associated with a lower capacity to be alone in wave 2. We also found reciprocal positive predictive relationships between depression and anxiety, and life satisfaction and self‐compassion. Life satisfaction mediated the relationship between self‐compassion and psychopathological variables (i.e., depression and anxiety). Additionally, self‐compassion mediated the association between life satisfaction and psychopathological variables and the association between capacity to be alone and psychopathological variables. Our study highlights the significance of early identification and intervention in depression and anxiety. We also discovered the possible self‐soothing function of self‐compassion as well as the importance of fostering positive personal characteristics.

## INTRODUCTION

Although the world has entered the post‐pandemic era, the outbreak of coronavirus has dramatically changed people's lives, affecting their physical and mental health and having a lasting influence (Xiong et al., 2020; Yıldırım et al., 2022). Mental health problems of college students have long been a concern, and the pandemic has exacerbated them (Tao, Niu, et al., 2023; Wang, Hou, et al., 2022), increased anxiety and depression (Wang et al., 2021) and decreased life satisfaction (Ooi et al., 2022). A meta‐analysis showed that the prevalences of anxiety and depression among college students increased from 38.9% to 40.7% and from 33.4% to 35.9%, respectively, after the pandemic (Li et al., 2022).

More specifically, the pandemic created an environment in which people had to stay at home, potentially leading to increased feelings of loneliness (Malcom, 2021; Thakur et al., 2023). Meanwhile, a considerable amount of research has indicated that solitude could contribute to mental disorders, including depression and anxiety (Milicev et al., 2023; Padmanabhanunni & Pretorius, 2022). Noticeably, as proposed by the diathesis‐stress model, the occurrence of mental health problems can be seen as the outcome of the combination of an adverse environment and personal characteristics (Zuckerman, 1999). Considering that the capacity to be alone and self‐compassion are two important personal characteristics that buffer against solitude, they may serve as protective factor, preventing individuals from suffering from depression and anxiety (Larson & Lee, 1996; Wang et al., 2023; Winnicott, 1958).

In this study, we used longitudinal data to investigate the intricate relationships between the aforementioned variables. It has to be pointed out that because the current study is based on college students, depression and anxiety mainly refer to the related symptoms and affects (such as anhedonia, nervousness, and sadness), rather than to clinically defined depression and anxiety. As a whole, this study will not only provide comprehensive insight into the impact of the pandemic but also help us recognize the protective factors that assist individuals in maintaining a good state of mental health in an adverse environment.

### The effect of the capacity to be alone and self‐compassion on depression and anxiety

Solitude is generally considered a negative factor, leading to detrimental effects such as loneliness, low self‐esteem, and anxious affect (Lin et al., 2020; Wang, 2016; White et al., 2022). However, if individuals can find comfort and engage in self‐reflection through solitude, or possess the capacity to be alone, solitude can have a positive impact (Larson & Lee, 1996). The capacity to be alone has been discussed as an indicator of emotional maturity. The capacity to be alone reflects an individual's ability to cope with solitude and renew their mental state (Larson & Lee, 1996; Winnicott, 1958), which may serve as a buffer against stress (Cimino & Cerniglia, 2021). Aligned with this, research has shown that a higher level of the capacity to be alone is negatively correlated with the levels of depression and anxiety (Lian et al., 2021). During the pandemic, many countries, including China, adopted quarantine measures that set individuals in unwilling solitude. During this period, individuals with a higher level of the capacity to be alone may have faced a lower risk of suffering from depression, anxiety, and social media overuse (Cimino & Cerniglia, 2021). According to this evidence, we propose our hypothesis 1:

Hypothesis 1 The prior capacity to be alone may have a negative impact on subsequent depression and anxiety.

Meanwhile, self‐compassion could also act as a factor protecting individuals against the detrimental effects of solitude (Wang et al., 2023). Self‐compassion is a concept rooted in eastern Buddhism and is defined as treating oneself with kindness, support, and understanding (Pauley & Mcpherson, 2010). Self‐compassion comprises three elements: self‐kindness, common humanity, and mindfulness, and involves being moved by one's own suffering and extending compassion toward oneself when facing negative life events (Chahar Mahali et al., 2021; Neff, 2003a; Neff, 2003b). Social mentality theory (SMT) proposes that different motivations for forming social relationships with others can direct different cognition, emotion, and behaviors (Liotti & Gilbert, 2011). This theory suggests that there is a threat system associated with insecurity, defensiveness, and the limbic system (Neff, Kirkpatrick, et al., 2007). Meanwhile, there is a self‐soothing system associated with secure attachment, safeness, and the oxytocin‐opiate system (Neff, Kirkpatrick, et al., 2007). Self‐compassion plays a significant role in human well‐being by deactivating the threat system and activating the self‐soothing system, thereby generating a sense of security and calmness and helping people cope with their environment (Barnett & Flores, 2016; Gilbert, 2005). Insecurity is often linked to depression and anxiety (Arenas et al., 2019; Cortés‐García et al., 2020), which can be seen as part of the threat system. Indeed, it is reasonable that self‐compassion may help to prevent and alleviate depression and anxiety, especially in stressful periods such as the pandemic (Liang et al., 2022). Aligned with the theory, a cross‐lagged study indicated that during the pandemic, adolescents with a higher level of self‐compassion were less likely to show symptoms of depression when experiencing increasing feelings of loneliness (Wang et al., 2023). Thus, we propose hypothesis 2:

Hypothesis 2 Prior self‐compassion may negatively influence subsequent depression and anxiety.

### The mediating role of life satisfaction

Life satisfaction, which is a crucial aspect of well‐being, refers to how individuals evaluate the quality of their lives across various domains (Hoseini‐Esfidarjani et al., 2022; Mamani‐Benito et al., 2022). The level of life satisfaction is significantly negatively correlated with anxiety and depression. A low level of life satisfaction may indicate dissatisfaction with basic psychological needs (Lataster et al., 2022). The basic psychological need theory proposes that frustration in fulfilling basic psychological needs may lead to increasing vulnerability in the face of a stressful environment, raising the risk for depression and anxiety (Vansteenkiste et al., 2020). Specifically, college students who experience a lower level of life satisfaction tend to report less happiness and more negative emotions, which may contribute to the appearance of mental disorders such as depression (Seo et al., 2018). However, the relationship between anxiety and life satisfaction is controversial. Most research has found that anxiety is negatively correlated with life satisfaction (Guney et al., 2010; Kumar, 2016; Tsitsas et al., 2019). However, another research has observed a positive relationship between anxiety and life satisfaction (Mamani‐Benito et al., 2022). This positive relationship was observed in a very unique group of former interns in the health sciences who were interning in specific situations (Mamani‐Benito et al., 2022). Thus, it requires caution to convey this result to a more general population, and further research is needed to better understand the relationship between anxiety and life satisfaction.

Meanwhile, previous research has declared that there is a robust positive relationship between self‐compassion and life satisfaction (Mantelou & Karakasidou, 2017; Terry & Leary, 2011). More specifically, high levels of self‐compassion have been linked to positive affect (Neff et al., 2007b), adaptive coping strategies (Ewert et al., 2021), and self‐care (Kim & Ko, 2018), all of which improve life evaluation and satisfaction. Apart from that, in reviewing previous studies, we found limited literature exploring the relationship between life satisfaction and the capacity to be alone. However, considering the theoretically positive side of the capacity to be alone (Larson & Lee, 1996), the capacity to be alone might provide individuals with an opportunity to assess the adverse environment and re‐establish emotional feelings, which may contribute to a higher level of happiness as well as a higher level of life satisfaction (Winnicott, 1958).

Based on the above, we propose the following hypotheses:

Hypothesis 3 Life satisfaction may mediate the relationship between self‐compassion and psychopathological variables (i.e., depression and anxiety).

Hypothesis 4 Life satisfaction may mediate the relationship between the capacity to be alone and psychopathological variables (i.e., depression and anxiety).

### The present study

Previous studies have the following limitations that need to be addressed. First, limited research has been conducted on positive variables such as self‐compassion, particularly regarding the capacity to be alone. Furthermore, while numerous studies have explored the relationships between the aforementioned variables, limited longitudinal research has been carried out, and further causal relationships require exploration. To address these limitations, our study employed longitudinal data from college students in Harbin, China, to delve deeper into the causal relationship between the aforementioned variables while testing the mediating role of life satisfaction.

To our knowledge, this is the first study to undertake a longitudinal analysis of the relationships and mechanisms between self‐compassion, the capacity to be alone, life satisfaction, depression, and anxiety. Furthermore, previous studies have suggested that age and gender may impact the prevalences of depression and anxiety (Tao et al., 2024), the level of self‐compassion (Yarnell et al., 2015), the relationship between self‐compassion and emotional well‐being (Bluth et al., 2017), and the relationship between self‐compassion and life satisfaction (Li et al., 2021). Hence, we controlled for the influence of age and gender by analyzing them as covariance variables in our research.

## METHOD

### Participants

This study was conducted in Harbin, China, with college students recruited to be participants. A total of 6710 college students participated in the first wave of data collection, and 3344 students in the same college participated in the second wave of collection. Some students only filled out the questionnaires collected in wave 1 or wave 2. After matching the student number for the two waves of data, we filtered out 1617 students who participated in both waves of the survey. After processing outliers, data from 1460 participants were finally included in the analysis. The mean age of these 1460 students was 18.60 years (*SD* = 1.31), with an age range of 16–28 years, and 56.51% were female.

### Procedure

In September 2021, COVID‐19 was widespread in Harbin, China, and higher education institutions were required to implement strict countermeasures, including restrictions on students' movements, to prevent the spread of the virus among college students. To measure the impact of these measures on college students' mental health, the first wave of data was collected in September 2021 (wave 1). A year later, in December 2022, the epidemic prevention measures were gradually relaxed, and students were allowed to enter the colleges and travel freely. At this time, a second wave of data (wave 2) was collected at the same colleges to assess the ongoing impact on students after repeated collective control measures. The data were collected through an online questionnaire (https://www.wjx.cn), and the link to the questionnaire was sent to students by college teachers. Participants were provided with an electronic version of the informed consent form and were informed of the purpose of the study prior to data collection. The study was approved by the Human Research Ethics Committee of Beijing Normal University (202112220084).

### Measures

#### Generalized Anxiety Disorder Scale (GAD‐7)

The 7‐item Generalized Anxiety Disorder Scale (GAD‐7) is used to measure the severity of anxiety symptoms (Spitzer et al., 2006). GAD‐7 is rated on a scale of 0 to 3, with a higher score indicating more severe symptoms. The total score ranges from 0 to 21. The questionnaire has good internal consistency among Chinese samples (Tao, Hou et al., 2023; Tao, Niu et al., 2023; Zhang et al., 2022). Cronbach's alpha values of GAD‐7 were 0.93 and 0.94 in waves 1 and 2, respectively.

#### Patient Health Questionnaire (PHQ‐9)

The Patient Health Questionnaire (PHQ‐9) is a 9‐item self‐report questionnaire used to measure the severity of symptoms of depression (Kroenke et al., 2001; Zhang et al., 2013). PHQ‐9 is graded on a scale of 0 to 3, with a higher score indicating more severe symptoms. The total score ranges from 0 to 27. The questionnaire has good internal consistency among Chinese samples (Tao et al., 2022; Wang, Hou, et al., 2022; Wang, Zhang, et al., 2022), with Cronbach's alpha values of 0.88 and 0.92 in waves 1 and 2, respectively.

#### The Self‐Compassion Scale‐Short Form (SCS‐SF)

Typically, self‐compassion is measured using the 26‐item Self‐Compassion Scale (SCS) (Neff, 2003a; Neff, 2003b), which evaluates self‐kindness, self‐judgment, common humanity, isolation, mindfulness, and over‐identification. In light of this, the Self‐Compassion Scale‐Short Form (SCS‐SF) was created (Raes et al., 2011) and revised in China (Hu, 2013), forming a simplified version of the SCS with only 12 items. Each item was measured on a five‐point Likert scale ranging from 1 (*hardly ever*) to 5 (*almost always*). The SCS‐SF has good psychometric properties, as evidenced by samples of college students (Babenko & Guo, 2019). In the present study, Cronbach's alpha reliability coefficients of this scale in waves 1 and 2 were 0.71 and 0.79, respectively.

#### Capacity to be Alone Scale (CAS)

A 20‐item questionnaire named the Capacity to be Alone Scale was used to measure an individual's ability to cope with stress in solitude (Larson, 1990). Chinese people have been widely surveyed using the Chinese translation of the revised CAS scale (Chen & Zhou, 2012; Wu & Chen, 2006). A five‐point Likert scale is utilized to evaluate each item, ranging from 1 (*strongly disagree*) to 5 (*strongly agree*). Nine items need to be reversed before calculating the total score. Higher scores imply a greater capacity to be alone. In the present study, Cronbach's alpha reliability coefficients of the CAS in waves 1 and 2 were 0.86 and 0.80.

#### Satisfaction with Life Scale (SWLS)

The Satisfaction with Life Scale (SWLS) gauges an individual's subjective assessment of life in general. Diener et al. (1985) created the SWLS, which has five components. Respondents rate each item on a seven‐point Likert scale: 1 is for *strongly disagree*, and 7 is for *strongly agree*. The SWLS has good psychometric properties (Pavot & Diener,^1993^) and high reliability when used with Chinese samples (Kong & You, 2013; Liang & Zhu, 2015). Cronbach's alpha for the SWLS was 0.92 in wave 1 and 0.95 in wave 2.

### Data analysis

All analyses were performed using R software (version 4.3.1; R Core Team, 2023) and Mplus (version 8.3; Muthén & Muthén, 2017). To start, we determined whether there was common method bias in the present study through Harman's single‐factor test. We also used attrition analysis to explore whether there were significant differences in demographic variables, anxiety, depression, life satisfaction, capacity to be alone, and self‐compassion between subjects who completed both measures and those who completed only the first wave. Second, we analyzed the descriptive information of the two waves of data and correlation statistics between different variables through the R package *psych* (Revelle, 2022). Third, we tested the longitudinal measurement invariance of the variables in the current study. Specifically, longitudinal measurement invariance was tested by placing incrementally stringent equality restraints upon model parameters, allowing comparisons of a configural, a metric, a scalar, and a strict model of life satisfaction, depression, and anxiety (Meredith, 1993). Additionally, self‐compassion and the capacity to be alone are second‐order variables. We tested the configural, first‐order metric, second‐order metric, first‐order scalar, and second‐order scalar longitudinal measurement invariance of self‐compassion and the capacity to be alone (Rudnev et al., 2018). We used the method of fitting‐index difference test to decide which invariance model of a variable was accepted (Meade et al., 2008; Zhu et al., 2023). In this analysis, the comparative fit index (CFI), the Tucker–Lewis index (TLI) were used to compare different types of invariance models and decided which invariance model was accepted. If the model fit did not differ significantly between models (△CFI <0.01, △TLI <0.01; Cheung & Rensvold, 1999), the model with the highest level of invariance was accepted. Fourth, we adopted cross‐lagged analysis to explore the longitudinal relationship between depression, anxiety, self‐compassion, capacity to be alone, and life satisfaction, taking gender and age as covariates. To further understand the mediating role of life satisfaction, we applied the structural equation model and the bootstrapping method (bootstrap = 5000) to examine the longitudinal mediation model (Preacher & Hayes, 2008). In this procedure, gender and age were controlled as covariates, and the procedure was conducted using the R package *lavaan* (Yves, 2012).

The following indexes were used to measure the goodness of the model: CFI, TLI, the root mean square error of approximation (RMSEA), and the standardized root mean square residual (SRMR). CFI and TLI should be higher than 0.90, and greater than 0.95 would be better. If RMSEA and SRMR are lower than 0.08, the model is well fitted (Hu & Bentler, 1999).

## RESULTS

### Attrition analysis

According to the attrition analysis, there were no significant differences in gender (*χ* ^2^ = 2.292, *df* = 1, *p = *.130). However, there were significant differences in age, depression, anxiety, self‐compassion, capacity to be alone, and life satisfaction between students who participated in the two wave collections and those who did not participate in the second wave of collection. The mean age of students who participated in two waves of collection was lower than that of those who participated only in the first wave of collection. Additionally, the mean levels of depression and anxiety among students who participated in the whole study were lower than those of the attrition students. In contrast, students who completed the two waves of collection had higher mean levels of self‐compassion, capacity to be alone, and life satisfaction. However, even though the *t*‐test results of these variables are significant, Cohen's *d* absolute values of the *t* test between the two groups are lower than 0.5. According to previous criteria, the Cohen's *d* absolute values of the current study represent small to moderate effect sizes (Cohen, 2016). This means that the differences between the attrition individuals and the individuals who completed the whole study may not be very large. The details *t*‐test results are shown in Table 1.

**TABLE 1:** Results of the attrition analysis.

Variables	t/χ^2^	*p*	Cohen's *d*
Gender	2.29	.130	
Age	23.34	<.001	0.55
Depression (wave 1)	16.46	<.001	0.37
Anxiety (wave 1)	20.06	<.001	0.41
Self‐compassion (wave 1)	−5.97	<.001	−0.15
Capacity to be alone (wave 1)	−3.18	.001	−0.10
Life satisfaction (wave 1)	−13.05	<.001	−0.34

### Shared method variance test

Our analysis showed that the first factor's interpretation rates—both with and without rotation—were lower than 40%, according to Harman's single‐factor test. No apparent common method bias was found in our study, according to the results (Podsakoff et al., 2003).

### Descriptive and correlation statistics

Table 2 presents the mean scores and standard deviations of each scale at two time‐points. Additionally, Table 2 shows the correlation coefficients between all variables. The analysis showed that females had higher levels of depression, self‐compassion, and capacity to be alone in waves 1 and 2. We also found that depression, anxiety, self‐compassion, and life satisfaction were significantly correlated with each other across time (*p* < .05). Specifically, depression and anxiety had a positive relationship, and they were both negatively correlated with self‐compassion and life satisfaction. Self‐compassion shared a positive association with life satisfaction. However, the capacity to be alone was only significantly correlated with self‐compassion (wave 2). Specifically, the analysis showed that the capacity to be alone was positively associated with self‐compassion (wave 2) (*r* = 0.21, *p* < .001).

**TABLE 2:** Means, standard deviations, and correlations between different variables of each scale at two time‐points.

Variable	M	SD	1	2	3	4	5	6	7	8	9	10	11
1. Gender^a^													
2. Age	18.60	1.31	−0.19***										
3. T1 Depression	1.19	0.25	0.07*	−0.11***									
4. T1 Anxiety	1.11	0.19	0.05	−0.11***	0.61***								
5. T1 Self−compassion	3.62	0.57	0.07**	0.08**	−0.33***	−0.29***							
6. T1 Capacity to be Alone	3.43	0.58	0.06*	−0.02	0.01	−0.03	0.11***						
7. T1 Life satisfaction	4.92	1.13	−0.03	0.06*	−0.30***	−0.26***	0.40***	0.08**					
8. T2 Depression	1.22	0.32	0.06*	−0.07**	0.34***	0.27***	−0.22***	0.02	−0.19***				
9. T2 Anxiety	1.15	0.28	0.04	−0.06*	0.27***	0.27***	−0.17***	−0.03	−0.15***	0.73***			
10. T2 Self−compassion	3.72	0.64	0.07**	0.05	−0.21***	−0.19***	0.39***	0.05	0.25***	−0.38***	−0.36***		
11. T2 Capacity to be Alone	3.44	0.58	0.06*	0.01	0.01	0.01	0.04	0.45***	−0.02	−0.01	−0.05	0.21***	
12. T2 Life satisfaction	5.00	1.22	−0.01	0.08**	−0.21***	−0.17***	0.24***	0.01	0.41***	−0.33***	−0.28***	0.44***	0.11***

*Note*: *M* and *SD* denote the mean and standard deviation, respectively.

^a^ Male = 1, female = 2.

* indicates *p* < .05;

** indicates *p* < .01;

*** Indicates *p* < .001.

### Longitudinal measurement invariance

The results of longitudinal measurement invariance are shown in Table 3. According to the results, the longitudinal measurement invariance of life satisfaction reached strict invariance. Anxiety reached partial metric invariance (free factor loading of item 5 and item 7). Depression reached the standard of metric invariance. Two two‐order variables, namely self‐compassion and the capacity to be alone, both achieved the standard of second‐order scalar invariance after modifying the configural models according to the modification index (details are shown in the Supplement).

**TABLE 3:** Longitudinal measurement invariance of capacity to be alone, self‐compassion, life satisfaction, depression, and anxiety.

Invariance tests	χ^2^	*df*	Model comparison	CFI	△CFI	TLI	△TLI	RMSEA	SRMR
Capacity to be alone									
M1 Configural invariance	4047.046	657		0.899		0.880		0.059	0.095
M2 First‐order Metric invariance	4076.055	675	M1–M2	0.898	0.001	0.883	−0.003	0.059	0.096
M3 Second‐order Metric invariance	4076.426	676	M2–M3	0.898	0.000	0.883	0.000	0.059	0.096
M4 First‐order scalar invariance	4228.684	694	M3–M4	0.894	0.004	0.881	0.002	0.059	0.096
M5 second‐order scalar invariance	4241.127	695	M4–M5	0.894	0.000	0.881	0.000	0.059	0.096
Self‐compassion									
M1 Configural invariance	1563.535	215		0.917		0.893		0.066	0.106
M2 First‐order metric invariance	1587.347	221	M1–M2	0.916	0.001	0.895	−0.002	0.065	0.107
M3 Second‐order metric invariance	1608.182	226	M2–M3	0.915	0.001	0.896	−0.001	0.065	0.108
M4 First‐order scalar invariance	1663.483	232	M3–M4	0.911	0.004	0.895	0.001	0.065	0.108
M5 Second‐order scalar invariance	1698.566	237	M4–M5	0.910	0.001	0.895	0.000	0.065	0.109
Life‐satisfaction									
M1 Configural invariance	130.973	29		0.985		0.977		0.049	0.023
M2 Metric invariance	138.583	33	M1–M2	0.984	0.001	0.979	−0.002	0.047	0.024
M3 Scalar invariance	179.968	37	M2–M3	0.979	0.005	0.974	0.005	0.051	0.026
M4 Strict Invariance	239.885	42	M3–M4	0.971	0.008	0.969	0.005	0.057	0.030
Depression									
M1 Configural invariance	369.39	125		0.933		0.918		0.037	0.040
M2 Metric invariance	402.31	133	M1–M2	0.926	0.007	0.915	0.003	0.037	0.049
M3 Scalar invariance	881.25	141	M2–M3	0.902	0.024	0.893	0.022	0.060	0.050
M4 Partial sclar invariance	804.082	133	M2–M4	0.911	0.015	0.898	0.017	0.059	0.052
Anxiety									
M1 Configural invariance	322.975	69		0.959		0.946		0.050	0.030
M2 Metric invariance	508.172	75	M1–M2	0.930	0.029	0.915	0.031	0.063	0.073
M3 Partial Metric invariance	368.955	73	M1–M3	0.952	0.007	0.940	0.006	0.053	0.041

Abbreviations: χ^2^ = chi square; *df* = degree of freedom; CFI = comparative fit index; TLI = Tucker‐Lewis index; RMSEA = root mean square error of approximation; SRMR = standardized root mean squared residual.

### Cross‐lagged model

**FIGURE 1:**
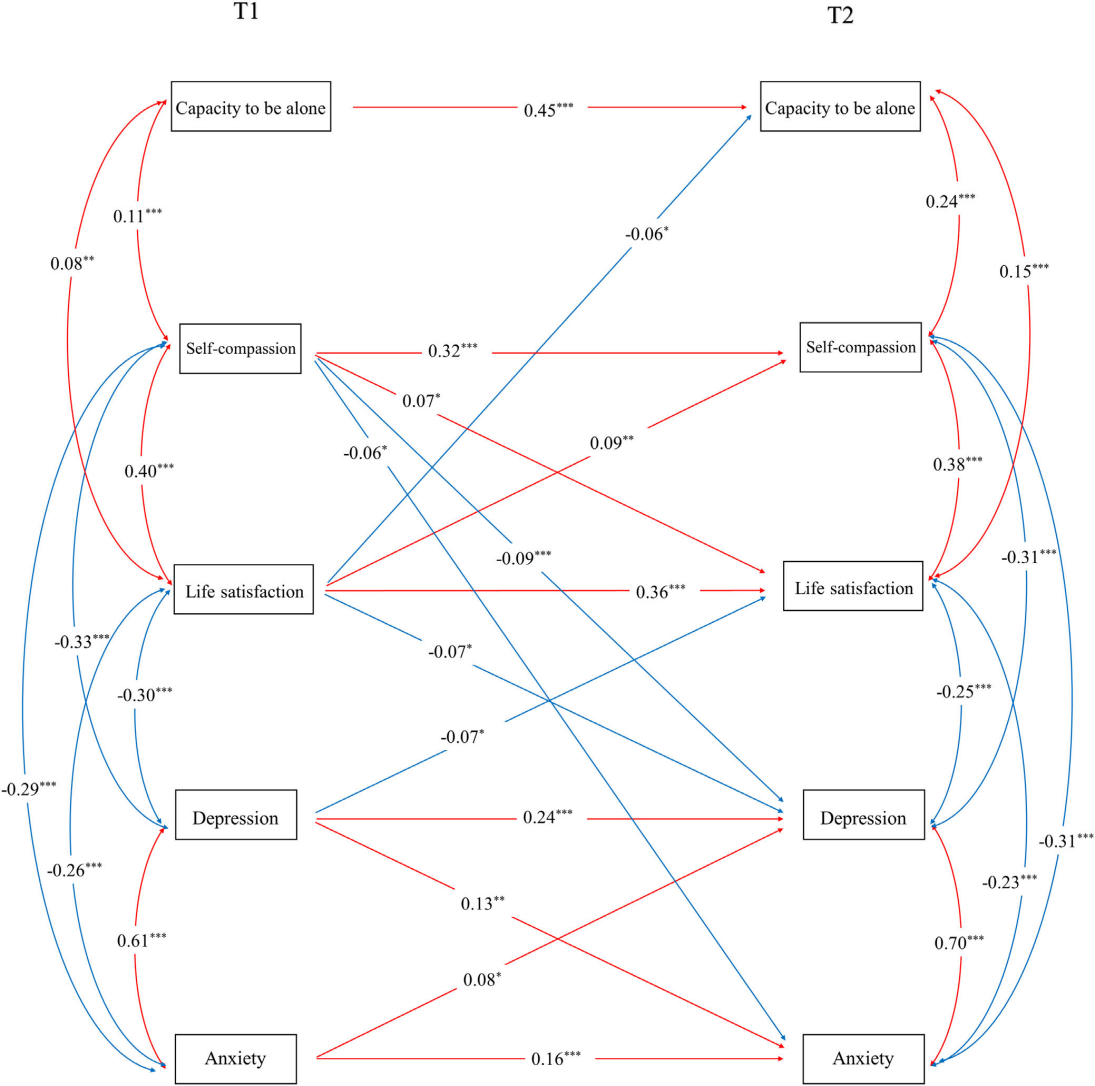
Cross‐lagged model. Note that “T1” indicates wave 1, and “T2” indicates wave 2. All the parameters are standardized. A red line represents a positive relationship, and a blue line represents a negative relationship. The final model with only significant paths is shown. * indicates *p* < .05. ** indicates *p* < .01. *** indicates *p* < .001.

As shown in Figure 1, the cross‐lagged model included depression, anxiety, self‐compassion, life satisfaction, and capacity to be alone, controlling for age and gender as covariates. The results showed that the model fitted the data well (*χ* ^2^ = 53.19, *df =* 10, CFI = 0.989, TLI = 0.930, SRMR = 0.028, RMSEA = 0.054). Our analysis showed that the autoregressive pathways of all variables were significant. This means that depression, anxiety, self‐compassion, capacity to be alone, and life satisfaction in wave 1 positively predicted the same variables in wave 2.

The cross‐lagged pathway showed that self‐compassion in wave 1 positively predicted life satisfaction in wave 2 (*B* = 0.149, *β* = 0.070, *p = *.017, 95% confidence interval [CI] [0.027, 0.274]) and negatively predicted depression (*B* = −0.052, *β* = −0.093, *p < *.001, 95% CI [−0.082, −0.023]) and anxiety (*B* = −0.031, *β* = −0.062, *p = *.018, 95% CI [−0.057, −0.006]) in wave 2. Life satisfaction in wave 1 negatively predicted depression (*B* = −0.019, *β* = −0.066, *p = *.019, 95% CI [−0.035, −0.003]) and capacity to be alone (*B* = −0.031, *β* = −0.061, *p = *.027, 95% CI [−0.058, −0.003]) in wave 2, while it positively predicted self‐compassion (*B* = 0.053, *β* = 0.094, *p = *.001, 95% CI [0.022, 0.085]) in wave 2. Furthermore, our results showed that life satisfaction and depression negatively and significantly predicted each other across two time‐points. Depression and anxiety positively predicted each other across time.

### Mediation analysis

The cross‐lagged analysis found that self‐compassion and life satisfaction positively predicted each other across two waves. Thus, we conducted longitudinal mediation models using self‐compassion and capacity to be alone as mediators, respectively. In Model 1 and Model 2, we examined whether life satisfaction in wave 1 (Model 1) and wave 2 (Model 2) could mediate the association between self‐compassion and capacity to be alone in wave 1 and depression and anxiety in wave 2, respectively. Because the current study has two waves of data, we compared Model 1 and Model 2 to test which wave of life satisfaction is more suitable to be the mediator. In Model 3 and Model 4, we analyzed the mediated effect of self‐compassion in wave 1 (Model 3) and wave 2 (Model 4) between life satisfaction and the capacity to be alone in wave 1 and depression and anxiety in wave 2, respectively. We also compared Model 3 and Model 4 to examine which wave of self‐compassion was more suitable to be the mediator. Gender and age were included as covariates in these models.

**FIGURE 2:**
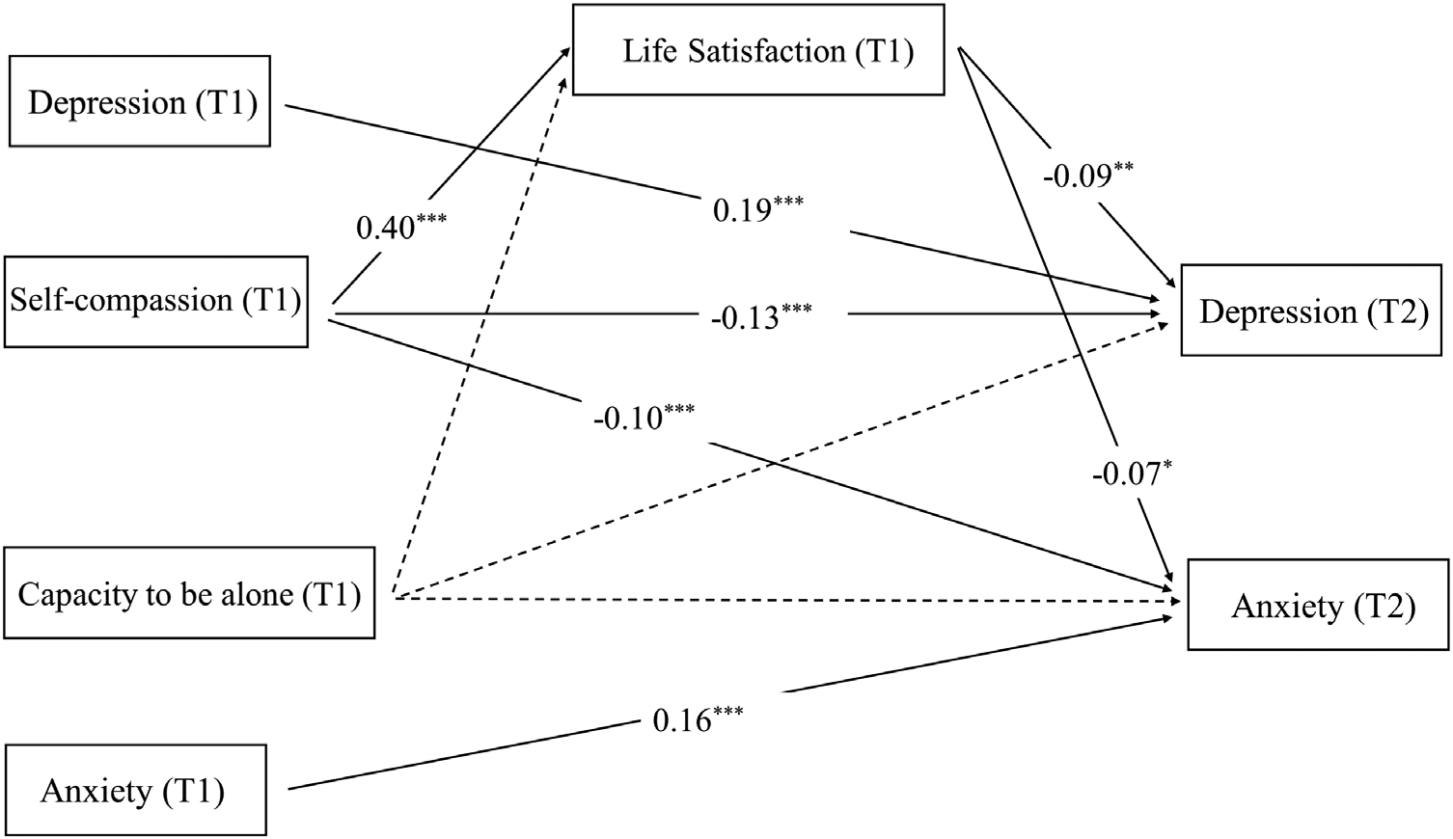
Model 1. Note that “T1” indicates wave 1, and “T2” indicates wave 2. All the parameters are standardized. A dashed line indicates no significant relationship, while a solid line indicates a significant relationship. * indicates *p* < .05. ** indicates *p* < .01. *** indicates *p* < .001.

The results of the goodness‐of‐fit indexes are shown in Table 4. Results show that the fitness of Model 1 (*χ* ^2^ = 87.326, *df* = 10, CFI = 0.970, TLI = 0.895, SRMR = 0.045, RMSEA = 0.073, Akaike information criterion [AIC] = 7772.661) and of Model 2 (*χ* ^2^ = 108.453, *df* = 10, CFI = 0.960, TLI = 0.861, SRMR = 0.054, RMSEA = 0.082, AIC = 8107.722) are acceptable. According to the criterion of comparing non‐nested models, the model with the lower AIC (Model 1) is better (West et al., 2012). In Model 1, life satisfaction (wave 1) mediated the relationship between self‐compassion (wave 1) and depression (wave 2), and the indirect effect was significant (*B* = −0.020, *β* = −0.036, *p = *.002, 95% CI [−0.033, −0.007]). The mediating effect of life satisfaction (wave 1) on the relationship between self‐compassion (wave 1) and anxiety (wave 2) was also significant (*B* = −0.013, *β* = −0.026, *p = *.028, 95% CI [−0.025, −0.001]). The results of Model 1 are depicted in Figure 2.

**TABLE 4:** Goodness‐of‐fit indexes for the mediation models.

Model	χ^2^	*df*	CFI	TLI	RMSEA	SRMR	AIC
Life satisfaction as mediator							
Model 1	87.326	10.000	0.970	0.895	0.073	0.045	7772.661
Model 2	108.453	10.000	0.960	0.861	0.082	0.054	8107.722
Self‐compassion as mediator							
Model 3	69.052	10.000	0.977	0.920	0.064	0.043	7754.387
Model 4	98.527	10.000	0.965	0.876	0.056	0.056	8220.663

Abbreviations: χ^2^ = chi square; AIC = Akaike information criterion; CFI = comparative fit index; RMSEA = root mean square error of approximation; SRMR = standardized root mean squared residual; TLI = Tucker–Lewis index. Model 1, Life satisfaction in wave 1 mediates the association between self‐compassion and capacity to be alone in wave 1 and depression and anxiety in wave 2; Model 2, Life satisfaction in wave 2 mediates the association between self‐compassion and capacity to be alone in wave 1 and depression and anxiety in wave 2; Model 3, Self‐compassion in wave 1 mediates the association between life satisfaction and the capacity to be alone in wave 1 and depression and anxiety in wave 2; Model 4, Self‐compassion in wave 2 mediates the association between life satisfaction and the capacity to be alone in wave 1 and depression and anxiety in wave 2.

**FIGURE 3:**
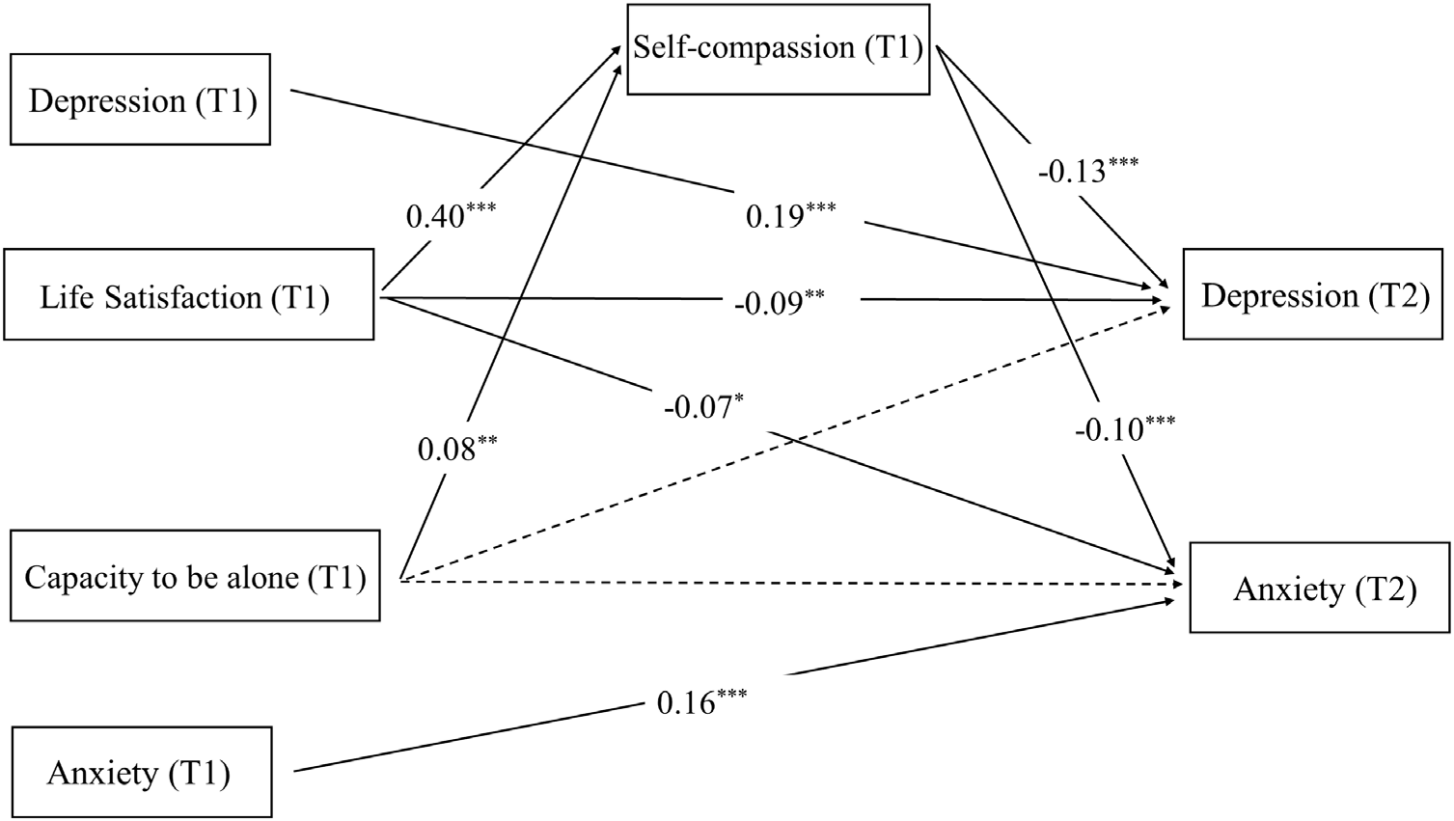
Model 3. Note that “T1” indicates wave 1, and “T2” indicates wave 2. All the parameters are standardized. A dashed line indicates no significant relationship, while a solid line indicates a significant relationship. * indicates *p* < .05. *** indicates *p* < .001. *** indicates *p* < .01.

The fitness of Model 3 is good (*χ* ^2^ = 69.052, *df* = 10, CFI = 0.977, TLI = 0.920, SRMR = 0.043, RMSEA = 0.064, AIC = 7754.387). The fitness of Model 4 is acceptable (*χ* ^2^ = 98.527, *df* = 10, CFI = 0.965, TLI = 0.876, SRMR = 0.056, RMSEA = 0.078, AIC = 8220.663). The comparison of AIC between Models 3 and 4 suggests that Model 3 is better. In Model 3, self‐compassion (wave 1) significantly mediated the association between life satisfaction (wave 1) and depression (wave 2) (*B* = −0.014, *β* = −0.049, *p < *.001, 95% CI [−0.020, −0.008]). Self‐compassion (wave 1) also served as a mediator between life satisfaction (wave 1) and anxiety (wave 2), with a significant indirect effect (*B* = −0.010, *β* = −0.039, *p < *.001, 95% CI [−0.015, −0.005]). The association between capacity to be alone (wave 1) and depression (wave 2) can be mediated by self‐compassion (wave 1) (*B* = −0.005, *β* = −0.010, *p = *.015, 95% CI [−0.010, −0.001]). Additionally, self‐compassion (wave 1) mediated the relation between capacity to be alone (wave 1) and anxiety (wave 2) (*B* = −0.004, *β* = −0.008, *p = *.024, 95% CI [−0.007, −0.001]). The results of Model 3 are depicted in Figure 3.

## DISCUSSION

In this research, we used longitudinal data from two time‐points to delve deeper into the complex relationship between self‐compassion, capacity to be alone, life satisfaction, depression, and anxiety, including gender and age as covariance variables in our model. Several important findings need to be discussed further.

Our cross‐lagged analysis uncovered relationships across time between different variables. Regarding the capacity to be alone, our study strikingly found that life satisfaction in wave 1 negatively predicted the capacity to be alone in wave 2. This result is inconsistent with what we expected and did not support Hypothesis 1. One possible explanation for this result is the background of COVID‐19. The second wave of data in the current study was collected in December 2022, and during this period, the government relaxed measures to control the pandemic (Liang et al., 2023). Thus, during the collection of wave 2 data, the number of COVID‐19 infections rose sharply, and students faced huge changes in their lives. This kind of major event during the pandemic may have affected their capacity to be alone, with previous research showing that public health emergencies and major events in life may have an association with individuals' loneliness (Firouzkouhi et al., 2023; Lasgaard et al., 2016). For example, the death of a loved one due to the pandemic might be related to the prolonged grieving, maladaptation, and loneliness of individuals (Firouzkouhi et al., 2023). Therefore, we infer that the negative relationship between life satisfaction (wave 1) and capacity to be alone (wave 2) in the current study might be due to the sharp changes in pandemic‐relevant policy during wave 2. Apart from this, the present study yielded that the capacity to be alone may decrease depression and anxiety through improving self‐compassion. This result is consistent with previous studies showing that capacity to be alone is an indicator of mental maturity and has a positive function in soothing psychopathological problems (Larson & Lee, 1996; Lian et al., 2021; Winnicott, 1958).

This research also showed that depression and anxiety could positively predict each other across time. A wealth of literature has identified the close relationship between depression and anxiety, suggesting that the comorbidity rate of depression and anxiety is high (Kalin, 2020; Saha et al., 2021). Huang and Liu (2023) conducted research in 2022, recruiting college students in China to evaluate their depression and anxiety, and found that the prevalence of depression‐anxiety comorbidity was 25.60%. These studies, together with the findings of our study, support that depression and anxiety have a close association. Additionally, given that the current study utilized longitudinal data, the results of this study also highlight the significance of early identification and intervention in depression or anxiety among college students. This can prevent the further development of the comorbidity of depression‐anxiety. Early identification and intervention in depression or anxiety can play a role in reducing the likelihood of detrimental outcomes caused by the comorbidity of depression‐anxiety (Groen et al., 2020).

Regarding the relationship between depression, anxiety, and life satisfaction shown in the cross‐lagged analysis, the results show that depression and life satisfaction had a negative predictive relationship over two time‐points, while anxiety and life satisfaction could not predict each other over two time‐points. The negative predictive relationship between depression and life satisfaction found in this research is in line with prior research (Joshanloo, 2022). However, the relationship between anxiety and life satisfaction identified in the current study is different from that in previous studies (Lin et al., 2021; Serin et al., 2010). One possible explanation for the finding is that the scores of the anxiety scale in the current study primarily reflect state anxiety among our participants, most of whom reported low levels of anxiety. Unlike trait anxiety, state anxiety is temporary and sensitive to changes (Pham et al., 2019) and may have different effects on life satisfaction (Tsitsas et al., 2019).

Additionally, our study was completed during the pandemic, and during this period, the local government's policy to prevent the spread of COVID‐19 often changed in response to the situation regarding the pandemic. Thus, the pandemic prevention policy would have had an important influence on the mood of college students (Chen et al., 2020). Wave 1 data in our study was collected in September 2021, during a lockdown period, which likely increased anxiety levels among students (Andersen et al., 2021). However, this kind of increase may not last, as research on longitudinal data shows that anxiety levels tend to rise and then fall during epidemics (Hettich et al., 2022; Zhao et al., 2022). These studies support our explanation that temporary state anxiety may not have long‐term effects on life satisfaction after 1 year.

The cross‐lagged analysis also discovered the mitigation effect of self‐compassion on depression and anxiety, with the results showing that a higher level of self‐compassion in wave 1 predicted lower levels of depression and anxiety in wave 2. This result supports Hypothesis 2 and is in line with multiple earlier studies that identified the buffer effect of self‐compassion on depression and anxiety (de Souza et al., 2020; Muris et al., 2016). These findings are consistent with the speculation of SMT and support that self‐compassion could deactivate the threat system and activate the self‐soothing system to mitigate depression or anxiety (Barnett & Flores, 2016; Gilbert, 2005; Liotti & Gilbert, 2011). Additionally, our analysis showed that self‐compassion and life satisfaction could promote each other over time. Not only did life satisfaction mediate the relationship between self‐compassion and psychopathological variables (i.e., depression and anxiety), but self‐compassion also mediated the relationship between life satisfaction and psychopathological variables (i.e., depression and anxiety). The results support Hypothesis 3 and do not support Hypothesis 4. Most studies have focused only on the effect of self‐compassion on life satisfaction, neglecting the possible effect of life satisfaction on self‐compassion (Mantelou & Karakasidou, 2017; Terry & Leary, 2011). However, some insights from psychological capital theory may explain the results of our study (Luthans & Youssef‐Morgan, 2017). Psychological capital is a psychological factor that leads to positive behaviors (Seligman, 2002), and is a kind of positive resource positively associated with life satisfaction (Datu & Valdez, 2019). Moreover, one study found that life satisfaction may have a significant positive predictive effect on psychological capital (Shan et al., 2022), which may suggest that life satisfaction can generate the development of psychological capital. Self‐compassion components are positively correlated with components of psychological capital (Sabaitytė & Diržytė, 2016). From the above evidence and our findings, we can infer not only that individuals with high levels of self‐compassion are more satisfied with their lives, but also that individuals who are more satisfied with their lives are more likely to develop higher levels of self‐compassion. Thus, improving college students' life satisfaction or self‐compassion may help to prevent and mitigate depression or anxiety.

There are some limitations in the present research that need to be acknowledged. First, as the study focused only on non‐clinical college students, caution is needed when generalizing the findings to other populations, especially to clinical populations. Second, the use of the GAD‐7 questionnaire to measure anxiety levels did not allow us to differentiate between state and trait anxiety, which limits the exploration of the specific mechanisms involved. Third, even though the present study utilized longitudinal data to explore the relationship between the capacity to be alone, self‐compassion, life satisfaction, depression, and anxiety, the two‐wave longitudinal study is only able to construct the half‐mediation model and it can not comprehensively reveal the causal relationship between variables. Future studies could collect three‐wave longitudinal data to further construct and test the mediation model between the aforementioned variables. Finally, the attrition analysis of the current study showed that significant differences existed between students who participated in the whole two‐wave collection and the lost subjects, even though the Cohen's *d* coefficients were small. Prudently, we suggest that the findings of this study may only be generalized to individuals who have milder symptoms of anxiety and depression and have higher levels of self‐compassion, life satisfaction, and the ability to be alone. Ascertaining whether the results can be generalized to other individuals needs more research.

## CONCLUSIONS

The present investigation used longitudinal data to explore the complex relationships between five variables (i.e., capacity to be alone, self‐compassion, life satisfaction, depression, and anxiety), yielding several noteworthy findings. The cross‐lagged analysis revealed that depression and anxiety significantly positively predict each other across time. Additionally, this kind of positive bidirectional predictive relationship was found between self‐compassion and life satisfaction. Second, we found that depression and life satisfaction could negatively predict each other across two time‐points. Third, this study discovered that self‐compassion in wave 1 negatively predicted depression and anxiety in wave 2, suggesting that self‐compassion was a protective factor of mental health. Fourth, in terms of the capacity to be alone, our study discovered that life satisfaction in wave 1 could negatively predict the capacity to be alone in wave 2. Finally, the examination of longitudinal mediation showed that life satisfaction in wave 1 could mediate the relationship between self‐compassion in wave 1 and mental health problems (i.e., depression and anxiety) in wave 2. Moreover, the mediating roles of self‐compassion in wave 1 between life satisfaction (wave 1) and mental health problems (wave 2) and between capacity to be alone (wave 1) and mental health problems (wave 2) were significant.

According to these findings, we highlight the significance of early identification and intervention in depression and anxiety among college students. Moreover, we propose that the cultivation of positive psychological characteristics (i.e., self‐compassion, capacity to be alone, and life satisfaction) would be helpful in preventing mental health problems.

## DISCLOSURE OF CONFLICT OF INTEREST

The authors declare there are no conflicts of interest.

## ETHICS STATEMENT

The authors assert that all procedures contributing to this work comply with the ethical standards of the relevant national and institutional committees on human experimentation and with the Helsinki Declaration of 1975, as revised in 2008. This research was examined and approved by the Ethical Committee of Beijing Normal University (202112220084).

## Supporting information


**Data S1:** Supporting Information.

## Data Availability

Data are available upon request from the first author.
