# Supplementary material for: Longitudinal associations between capacity to be alone, life satisfaction, self‐compassion, anxiety, and depression among Chinese college students
Source: Psych J. 2024 Jun 23;13(6):979–92. doi: 10.1002/pchj.783 (PMC11608793; doi:10.1002/pchj.783)
Supplement: Supplementary file 1 — Data S1: Supporting Information. [file PCHJ-13-979-s001.docx]

Supplement 1. The Mplus code of testing the configural invariance of capacity to be alone

DATA: FILE = InvarianceDatamplusO.DAT;

VARIABLE: NAMES ARE Gender Age T1O1-T1O20 T2O1-T2O20; !T1O1-T1O20 are 20 items of capacity to be alone in wave 1 and T2O1-T2O20 are items in wave 2

USEVARIABLE ARE T1O1-T1O20 T2O1-T2O20;

ANALYSIS: ESTIMATOR=MLM;

MODEL:

T1V1 BY T1O1 T1O2 T1O3 T1O4 T1O5 T1O6 T1O7 T1O8 T1O9 T1O10;!T1V1 is the first dimension (first-order factor) of Capacity to be Alone Scale (CAS) in wave 1.

T1V2 BY T1O11 T1O12 T1O13 T1O14 T1O15 T1O16 T1O17 T1O18 T1O19 T1O20; !T1V2 is the second dimension (first-order factor) of CAS in wave 1.

T1O BY T1V1 T1V2; !T1O is the second-order factor of CAS in wave 2.

T1O13 WITH T1O14;

T1O19 WITH T1O20;

T1O16 WITH T1O17;

T1O12 WITH T1O11;

T1O9 WITH T1O7;

T1O2 WITH T1O1;

T1O10 WITH T1O9;

T1O7 WITH T1O3;

T1O9 WITH T1O3;are

T1O16 WITH T1O15;

T1O16 WITH T1O14;

T1O17 WITH T1O15;

T1O16 WITH T1O13;

T1O17 WITH T1O14;

T1O17 WITH T1O13;

T1O15 WITH T1O13;

T1O15 WITH T1O14;

T1O10 WITH T1O6;

T1O18 WITH T1O19;

T1O18 WITH T1O20;

T1O4 WITH T1O8;

T1O11 WITH T1O15;

T1O8 WITH T1O1;

T1O10 WITH T1O3;

T1O10 WITH T1O7;

T1O3 WITH T1O2;

T1O5 WITH T1O3;

T1O8 WITH T1O2;

T1O10 WITH T1O4;

T2V1 BY T2O1 T2O2 T2O3 T2O4 T2O5 T2O6 T2O7 T2O8 T2O9 T2O10;

T2V2 BY T2O11 T2O12 T2O13 T2O14 T2O15 T2O16 T2O17 T2O18 T2O19 T2O20;

T2O BY T2V1 T2V2;

T2O13 WITH T2O14;

T2O19 WITH T2O20;

T2O16 WITH T2O17;

T2O12 WITH T2O11;

T2O9 WITH T2O7;

T2O2 WITH T2O1;

T2O10 WITH T2O9;

T2O7 WITH T2O3;

T2O9 WITH T2O3;

T2O16 WITH T2O15;

T2O16 WITH T2O14;

T2O17 WITH T2O15;

T2O16 WITH T2O13;

T2O17 WITH T2O14;

T2O17 WITH T2O13;

T2O15 WITH T2O13;

T2O15 WITH T2O14;

T2O10 WITH T2O6;

T2O18 WITH T2O19;

T2O18 WITH T2O20;

T2O4 WITH T2O8;

T2O11 WITH T2O15;

T2O8 WITH T2O1;

T2O10 WITH T2O3;

T2O10 WITH T2O7;

T2O3 WITH T2O2;

T2O5 WITH T2O3;

T2O8 WITH T2O2;

T2O10 WITH T2O4;

T1O1-T1O20 PWITH T2O1-T2O20;

OUTPUT: STANDARDIZED;

Supplement 2. The Mplus code of testing the configural invariance of self-compassion

DATA: FILE = InvarianceDatamplusE.DAT;

VARIABLE: NAMES ARE Gender Age T1E1-T1E12 T2E1-T2E12; ! T1E1-T1E12 are 12 items of self-compassion in wave 1 and T2E1-T2E12 are items in wave 2

USEVARIABLE ARE T1E1-T1E12 T2E1-T2E12;

ANALYSIS: ESTIMATOR=MLM;

MODEL:

T1SK BY T1E2 T1E6;

T1MF BY T1E3 T1E7;

T1CH BY T1E5 T1E10;

T1SJ BY T1E11 T1E12;

T1IS BY T1E4 T1E8;

T1OI BY T1E1 T1E9; ! T1SK T1MF T1CH T1SJ T1IS T1OI are six dimensions (first-order factors) of The Self-Compassion Scale-Short Form (SCS-SF)

T1E2 WITH T1E3;

T1E7 WITH T1E6;

T1E4 WITH T1E1;

T1E9 WITH T1E4;

T1E9 WITH T1E8;

T1E8 WITH T1E1;

T1E BY T1SK T1MF T1CH T1SJ T1IS T1OI; ! T1E is the second-order factor of SCS-SF in wave 1

T2SK BY T2E2 T2E6;

T2MF BY T2E3 T2E7;

T2CH BY T2E5 T2E10;

T2SJ BY T2E11 T2E12;

T2IS BY T2E4 T2E8;

T2OI BY T2E1 T2E9;

T2E2 WITH T2E3;

T2E7 WITH T2E6;

T2E4 WITH T2E1;

T2E9 WITH T2E4;

T2E9 WITH T2E8;

T2E8 WITH T2E1;

T2E BY T2SK T2MF T2CH T2SJ T2IS T2OI;

T1E1-T1E12 PWITH T2E1-T2E12;

OUTPUT: STANDARDIZED;
